# Fatal European subtype tick-borne encephalitis in a fully vaccinated immunocompetent child: a case report with viral sequencing

**DOI:** 10.1186/s12879-026-13657-0

**Published:** 2026-05-29

**Authors:** Klara Sondén, Oskar Karlsson Lindsjö, Sofie Vonlanthen, Magnus Gisslén, Amelie Kinch

**Affiliations:** 1 Division of Infectious Diseases, Department of Medicine Solna, Karolinska Institute, Stockholm, Sweden; 2 Department of Microbiology, Public Health Agency of Sweden, Stockholm, Sweden; 3 Department of Clinical Immunology and Transfusion Medicine, Karolinska University Hospital, Stockholm, Sweden; 4 Department of Laboratory Medicine, Division of Clinical Immunology, Karolinska Institute, Stockholm, Sweden; 5 Department of Infectious Diseases, Institute of Biomedicine, Sahlgrenska Academy at University of Gothenburg, Gothenburg, Sweden; 6 Department of Infectious Diseases, Sahlgrenska University Hospital, Region Västra Götaland, Gothenburg, Sweden; 7 Department of Medical Sciences, Section of Infectious Diseases, Uppsala University, Uppsala, S-751 85 Sweden

**Keywords:** TBE, European subtype, Fulminant encephalitis, Paediatric, Viral sequencing, TBEV NS1 antigen

## Abstract

**Background:**

Tick-borne encephalitis (TBE) is considered to have a more favourable outcome in children compared to adults, and death from TBE in children is infrequent. Severe cases are primarily seen in immunocompromised or unvaccinated individuals. We report a case of TBE with a fatal outcome in a previously healthy child. The case is unique in several aspects: an atypical clinical presentation without fever or biphasic illness, and a fulminant course despite full vaccination and absence of known predisposing risk factors for severe disease. In addition, sequencing revealed the TBE virus (TBEV) as a previously identified European subtype covered by the vaccine.

**Case presentation:**

The 9-year-old child had an acute onset of headache but no fever. On the fourth day, the child was lethargic but responded adequately to questions and was sent home after assessment at the Emergency Department (ED). The next day, the child returned to the ED, where four generalised tonic-clonic seizures were observed. Analysis of cerebrospinal fluid showed lymphocytic pleocytosis consistent with viral encephalitis. A computed tomography scan showed general oedema of the brain, hydrocephalus, and incipient herniation. Intracranial pressure remained extremely high despite hyperventilation with mechanical ventilation, mannitol, thiopental, bilateral ventricular drainage, and methylprednisolone. Four-vessel angiography on day 10 showed a picture consistent with brain death. No immunodeficiency was identified by whole-genome sequencing. The aetiology of the encephalitis was determined by detection of IgG antibodies against the TBEV non-structural protein 1 (NS1) antigen (which is not present in existing vaccines) and by post-mortem PCR of brain tissue. A previously unpublished tiled amplicon method was used to recover the TBEV genome, which was assigned to the European subtype (TBEV-Eur).

**Conclusions:**

This case report highlights the risk of delayed diagnosis when atypical symptoms are present. TBE should be considered when investigating causes of headaches in highly endemic areas, even if the patient lacks fever or is vaccinated. Further, since children may also develop very severe disease, it is important to vaccinate children against TBE. Finally, analysis of antibodies to TBE NS1 antigen is a valuable tool to diagnose TBE in vaccinated individuals.

**Supplementary Information:**

The online version contains supplementary material available at 10.1186/s12879-026-13657-0.

## Background

Tick-borne encephalitis (TBE) is caused by the TBE virus (TBEV) and transmitted primarily by tick bites. There are three main subtypes of TBEV, namely the European, Siberian, and Far-eastern subtypes. Recently, two additional subtypes were identified, the Himalayan and Baikalian subtypes [1]. The European subtype of TBE is endemic in southern Sweden, and the incidence has increased continuously for over a decade, reaching about 5 per 100,000 inhabitants in Sweden and 8–12 per 100,000 in Uppsala County, where this case occurred [2, 3]. TBE is also increasing in other European countries and globally [4]. The other subtypes are mainly found in Russia and Asia and have not been identified in Sweden [5]. However, the Russian subtype has been detected in our neighbouring country Finland.

Infection with the European subtype of TBEV is often asymptomatic (around 75% of cases) or mild, and only 10-30% of adults develop infection of the central nervous system (CNS). In symptomatic patients, there is typically (seen in two thirds of the patients) a biphasic clinical course with an initial febrile phase, followed by a symptom-free interval of about one week, and then return of fever and debut of neurological symptoms [6]. The infection of the CNS ranges from mild meningitis to severe encephalitis, and more seldom myelitis [6, 7]. Mortality rates in TBE infection in adults are reported to be 0.5–2% for the European subtype as compared to 1–3% for the Siberian, and up to 35% for the Far-Eastern subtypes [6, 7].

In the past, it was believed that children did not contract severe forms of TBE infection, but this notion has been challenged in recent years [1, 8]. Approximately 5–30% of children develop CNS infection and there is growing evidence that also children may experience long-term sequelae [1, 5, 7, 9]. Meningitis is the most common form of CNS manifestation in children (approximately 60–80%), followed by meningoencephalitis (approximately 15–40%) [1, 9]. Several studies report that children aged eight years and older tend to have a more severe clinical course than younger children [10, 11]. Children are reported to have more favourable outcomes than adults, and deaths in children are extremely rare (0.2%) [1, 12, 13].

There are two approved TBE vaccines available in Europe [14]. The overall seroconversion rates and effectiveness of the vaccines are very high both in adults and children, although the effectiveness is reported to be somewhat lower in children under the age of 15 compared to adults [15, 16]. In an Austrian study the field effectiveness was 97% in children younger than 15 years and remained above 90% in irregularly vaccinated children [15]. When the first vaccine was introduced in Sweden almost 40 years ago, children were not recommended for TBE vaccination since they were considered to have a low risk of developing severe disease. For a couple of years, vaccination has not only been recommended but also provided free of charge to children in the highly endemic area of Sweden where this case occurred.

Here we report a case of fulminant encephalitis caused by TBE in a previously healthy child. This case is highly unusual, given that the child was fully vaccinated, immunocompetent, and yet experienced a fatal outcome. The TBE genome was sequenced and found to be of the European subtype.

## Case presentation

### Clinical presentation

**Fig. 1:**
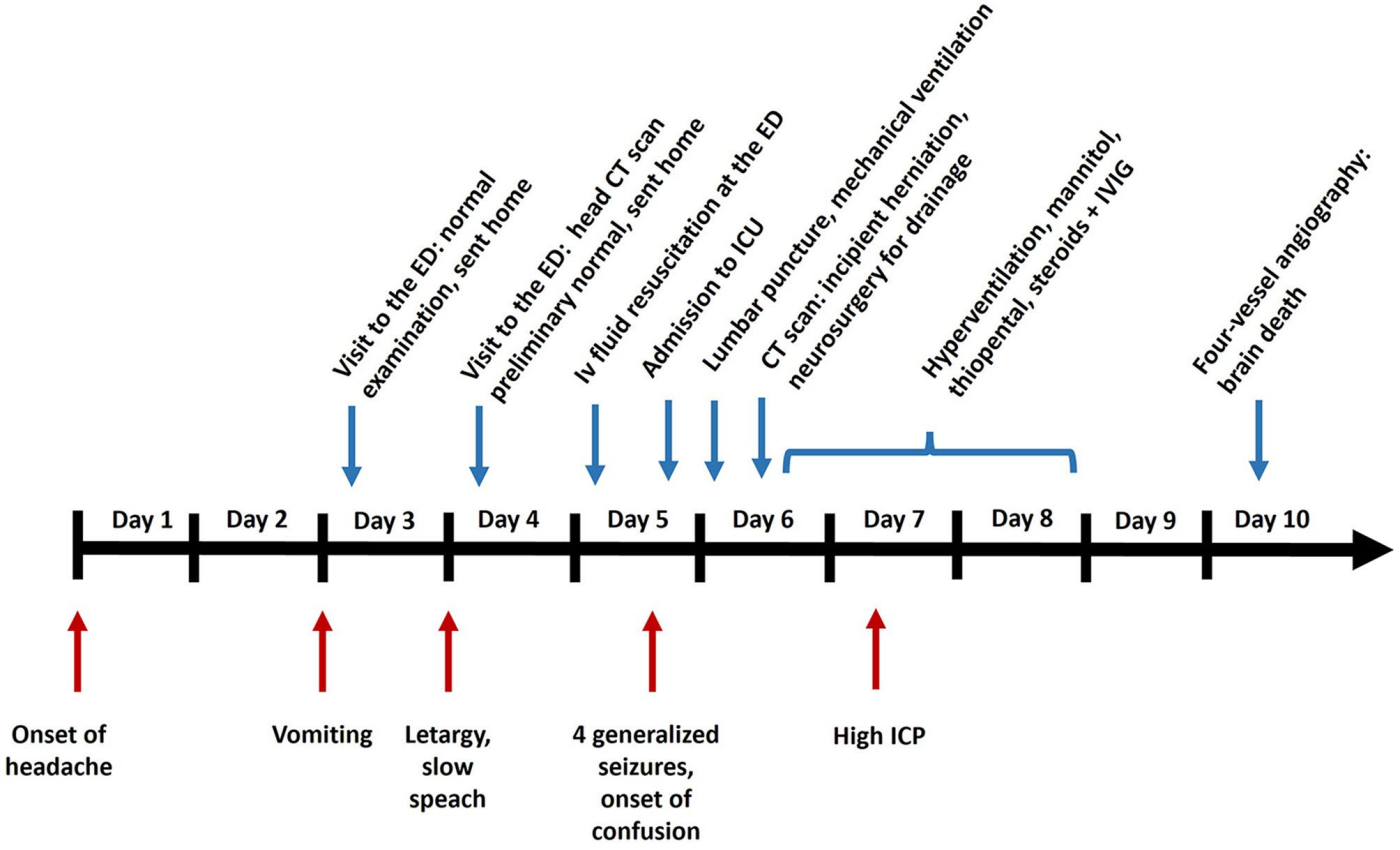
A timeline of the clinical course from the first symptom of encephalitis to death. Symptoms are presented below the line. Medical investigations and treatment of TBE and high intracranial pressure are shown above the line. Abbreviations: ED, Emergency department, CT, computed tomography, ICU, intensive care unit, ICP, intracranial pressure, IVIG, intravenous immunoglobulins

The clinical course is summarised in a timeline in Fig. 1. The 9-year-old, previously healthy child, active in several sports and rarely ill, developed a headache in early October. There were no fever or focal symptoms. On the third day of headache, the child woke up during the night and vomited. The headache decreased when lying down and was somewhat relieved by paracetamol. On day 3, the child was examined at the Paediatric Emergency Department (ED) at Uppsala University Hospital, Sweden. General physical examination, including neurological examination, was normal. The condition was assessed as migraine, and the child was sent home with a recommendation to take ibuprofen. There was a heredity for migraines.

On day 4, the family sought care at the ED again since the child’s general condition had deteriorated. The headache had worsened, with increased nausea and vomiting, and the child had become lethargic and had slow speech. However, the child was oriented to time, place, and person and responded adequately to questions. Physical examination was normal apart from lethargy and slow speech. There was still no fever and no neck stiffness. When the patient fell asleep, apnoeas were observed. Serum C-reactive protein (CRP) was 28 mg/L, haemoglobin (Hb) level was 112 g/L, white blood cell (WBC) count was 18.9 × 10^9^ cells/L, and neutrophils 16.7 × 10^9^ cells/L. Blood cultures drawn on this day were negative. A computed tomography (CT) scan of the brain was performed and was preliminarily assessed as normal. Later reassessment of the CT scan during office hours showed slightly dilated ventricles. The patient was sent home with a scheduled revisit to the ED for a lumbar puncture the following day.

On day 5, the child was subfebrile and sensitive to light on return at the ED. The headache had varied in intensity throughout the night, but never completely ceased. The paediatrician on call suspected that the patient was dehydrated and prescribed intravenous (IV) fluid replacement therapy with 12.5 ml/kg/h for four hours. With a body weight of 40 kg, he received 2 litres of IV fluids over the next few hours. In the evening, the child presented with a sudden generalised tonic-clonic seizure, resolved by midazolam, and had four similar episodes altogether during two hours. The seizures ceased after levetiracetam had been administered. Between the seizures, the child was continuously confused and hallucinating. The patient was admitted to the paediatric intensive care unit. After midnight on day 6, a lumbar puncture was performed. Opening pressure was not measured. There was a cerebrospinal fluid (CSF) pleocytosis with a predominance of mononuclear white blood cells (331×10^6^/L) and a slight rise also in polymorphonuclear leukocytes (54×10^6^/L). There was no sign of glucose consumption in CSF (glucose level 5.6 mmol/L in CSF, 7.6 mmol/L in plasma), the CSF albumin level was 454 mg/L, and CSF lactate level was 3 mmol/L. This analysis was consistent with viral encephalitis.

**Fig. 2:**
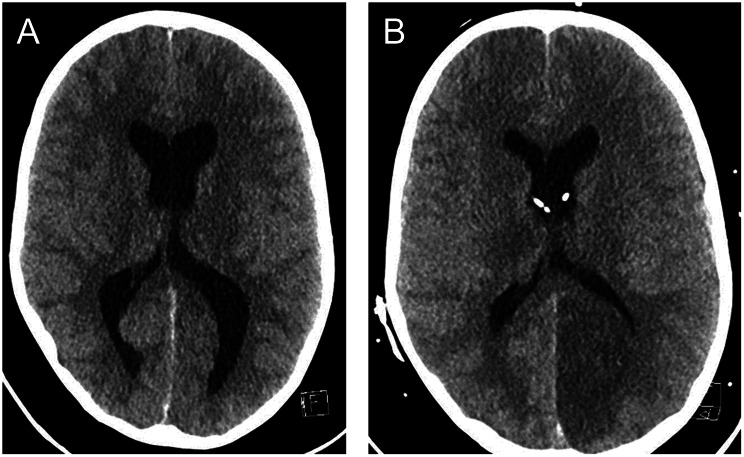
(**A**) a CT scan of the brain on day 6 showed general oedema, hydrocephalus, and incipient herniation. External ventricular drainages were urgently inserted bilaterally. (**B**) a postoperative CT scan showed residual mass effect, herniation, and development of multiple infarctions

The child was sedated with propofol before the lumbar puncture and did not wake up during the night towards day 6. Suddenly, the monitors of the patient’s vital signs alerted to hypoxia, and the child was urgently intubated and put on a mechanical ventilator. An acute CT scan of the brain performed on the morning of day 6 showed general oedema of the brain, hydrocephalus, and incipient herniation (Fig. 2A). Dilated pupils bilaterally were noted. Treatment with hyperventilation, mannitol, and thiopental was started, and the patient was immediately accepted for neurosurgery. Intracranial pressure (ICP) was elevated, measured at approximately 40–50 mm Hg intraoperatively. A catheter was first inserted into the right ventricle, but because a postoperative CT scan showed that the ventricles remained wide, the patient also received a catheter on the left side. In addition, an intraparenchymal catheter for pressure monitoring was inserted. Postoperatively, ICP was normal (15 mm Hg). Treatment with hyperventilation and thiopental coma was continued. Treatment with Methylprednisolone (30 mg/kg daily for three days) and intravenous immunoglobulins (IVIG, Octagam, 0.75 g/kg as a single dose) was administered. Later on day 6, a new CT scan of the brain showed residual mass effect, herniation, and development of multiple infarctions (Fig. 2B).

On day 8, no more CSF were drained from the external ventricular drains, and ICP was extremely high (70–120 mm Hg). The possibility of recovery was assessed as non-existent, and therefore thiopental was discontinued on day 9. Four-vessel angiography on day 10 showed no intracerebral blood circulation, which is consistent with brain death. Thus, all life-sustaining measures were suspended. An autopsy was performed.

### Microbiology and treatment of infection

After the seizures on the evening of day 5, the patient was given cefotaxime and aciclovir for meningoencephalitis of unknown origin. Multiplex PCR for respiratory virus in nasopharyngeal swab on day 6 was positive for rhinovirus, whereas it was negative for the other 14 tested viruses (Table 1). Rhinovirus PCR in CSF was negative. The meningitis/encephalitis PCR panel in CSF was positive for Human herpes virus 6 (HHV-6) and negative for the other 13 tested agents (Table 1). Antiviral treatment was switched from aciclovir to ganciclovir on day 6 to cover HHV-6, although it was considered unlikely that HHV-6 was the cause of the encephalitis. Confirmatory samples were sent to a reference laboratory, where HHV-6 PCR in CSF was negative, whereas low levels of HHV-6 DNA were detected in plasma. This was interpreted as a subclinical reactivation occurring during another severe disease. Ganciclovir was discontinued on day 9. Both PCR and serology for herpes simplex and varicella zoster viruses were negative. Cefotaxime was discontinued after three days, following negative CSF culture and serology for Borrelia burgdorferi.

**Table 1:** Results of the microbiological investigations (excluding TBE) in alphabetical order

Microbiological agent	Material	Method	Test result	Sample day
Adenovirus	NPS	Multiplex PCR	Negative	6
	CSF	PCR	Negative	9
	Plasma	PCR	Negative	9
Bacteria	CSF	Culture	Negative	6
*Borrelia burgdorferi*	CSF	Serology	Negative	6
	Serum	Serology	Negative	6
CMV	CSF	PCR (FilmArray)	Negative	6
	Serum	Serology	IgG positive, IgM negative	9
	Plasma	PCR	Negative	9
Coronaviruses OC43, HKU1, and NL63	NPS	Multiplex PCR	Negative	6
*Cryptococcus neoformans/gattii*	CSF	PCR (FilmArray)	Negative	6
EBV	Serum	Serology	IgG positive, IgM negative	9
	Plasma	PCR	Negative	9
Enterovirus	CSF	PCR (FilmArray)	Negative	6
	CSF	PCR	Negative	6
	NPS	PCR	Negative	6
*Escherichia coli*	CSF	PCR (FilmArray)	Negative	6
*Hemophilus influenzae*	CSF	PCR (FilmArray)	Negative	6
HHV-6	CSF	PCR (FilmArray)	Positive	6
	CSF	PCR (reference laboratory)	Negative	6
	Plasma	PCR (reference laboratory)	Positive, low levels	6
HIV	Serum	Serology	Negative	6
	Serum	Antigen test	Negative	6
HMPV	NPS	Multiplex PCR	Negative	6
HSV-1 and 2	CSF	PCR (FilmArray)	Negative	6
	Serum	Serology	IgG positive, IgM negative	9
	CSF	Serology	Negative	9
Influenza A and B	NPS	Multiplex PCR	Negative	6
*Listeria monocytogenes*	CSF	PCR (FilmArray)	Negative	6
Mycobacteria	CSF	Culture	Negative	9
	CSF	PCR	Negative	9
*Mycoplasma pneumoniae*	NPS	PCR	Negative	9
	Serum	Serology	IgG positive, IgM negative	9
*Neisseria meningitidis*	CSF	PCR (FilmArray)	Negative	6
Parainfluenza 1–4	NPS	Multiplex PCR	Negative	6
Parechovirus	CSF	PCR (FilmArray)	Negative	6
Parvovirus	Plasma	PCR	Negative	9
Rickettsia	CSF	PCR	Negative	6
Rhino/enterovirus	NPS	Multiplex PCR	Positive	6
Rhinovirus	NPS	PCR	Positive	6
	CSF	PCR	Negative	6
RSV	NPS	Multiplex PCR	Negative	6
SARS-CoV-2	NPS	Multiplex PCR	Negative	6
	Serum	Serology	IgG positive	9
*Streptococcus agalactiae*	CSF	PCR (FilmArray)	Negative	6
*Streptococcus pneumoniae*	CSF	PCR (FilmArray)	Negative	6
VZV	CSF	PCR (FilmArray)	Negative	6
	Serum	Serology	IgG positive, IgM negative	9
	CSF	Serology	Negative	9
West Nile virus	CSF	PCR	Negative	9
	CSF	Serology	Negative	9
	Plasma	PCR	Negative	9
	Serum	Serology	Negative	9

Abbreviations: NPS, nasopharyngeal swab, PCR, polymerase chain reaction, CSF, cerebrospinal fluid, CMV, Cytomegalovirus, EBV, Epstein-Barr virus, HHV-6, Human herpes virus 6, HIV, Human immunodeficiency virus, HMPV, Human metapneumovirus, HSV, Herpes simplex virus, RSV, Respiratory syncytial virus, SARS-CoV-2, Severe acute respiratory syndrome coronavirus 2, VZV, Varicella-zoster virus

Clinically, TBE was suspected since the patient lived in a highly endemic area for TBE in Sweden and had had several tick bites during the summer and autumn. The child had received primary immunisation against TBE with three vaccine doses, starting 3.5 years earlier, with the third dose administered about three years earlier. The routine TBE serology (Enzyme Immunoassay Test) on day 6 was positive for IgG, but negative for IgM, which neither confirms nor excludes the diagnosis of TBE in a vaccinated individual. For that reason, TBE PCR in CSF was performed, but was negative. Then, another TBE serology, the Suspension Multiplex Immunoassay (SMIA), which may differ between antibodies induced by infection and those induced by vaccination, was performed on blood samples. This method measures antibodies against both the whole virus and the TBE virus non-structural protein 1 (NS1) antigen that is not present in existing TBE vaccines [17]. In this TBE SMIA serology, IgG antibodies against the TBE NS1 antigen were detected, supporting the diagnosis of TBE (Table 2). At autopsy, a brain parenchymal biopsy was sent for TBE PCR, which was positive for two PCR target genes (NSE-3 and COMPLEX) with a cycle threshold of 27, confirming the diagnosis of TBE.

**Table 2:** Investigations for tick-borne encephalitis (TBE)

Material	Method	Test result	Sample day
Serum	Serology (Enzyme Immunoassay Test)	IgG positive, IgM negative	6
Serum	Serology (Suspension Multiplex Immunoassay)	IgG positive and IgM negative for whole virus. IgG positive and IgM negative for NS1-antigen.	6 and 9
CSF	PCR	Negative	6
Brain tissue	PCR	Positive	From autopsy

Abbreviations: NS1, non-structural protein 1, CSF, cerebrospinal fluid, PCR, polymerase chain reaction

While waiting for the results of the TBE serology (SMIA), other causes of encephalitis, such as human immunodeficiency virus, rickettsia, Epstein-Barr virus, parvovirus, mycoplasma, mycobacteria, and West Nile fever, were ruled out (Table 1).

### Other medical evaluation

Neuronal antibodies were not detected in serum or CSF. Kidney and liver function tests were normal. Serum protein electrophoresis showed mild to moderate inflammation, with normal immunoglobulin levels and no M-protein. Blood samples showed no sign of inborn errors of metabolism. An extensive genetic panel for immunodeficiency was performed, but showed no abnormal results (see more details in the next paragraph).

### Human genetic analyses

Whole-genome sequencing (WGS) of the patient was performed on a blood sample collected on day 10 to investigate a possible genetic cause of the severe clinical course (Supplementary Tables S1-S12). Sequencing and bioinformatic analysis were performed as previously described at Clinical Genomics, SciLife Lab, Stockholm, Sweden [18]. Due to low DNA concentration of the sample, the WGS library was performed using a low-input protocol (NxSeq® AmpFREE Low DNA Library Kit, Lucigen). Genetic variant detection was based on a gene panel for Primary Immunodeficiency, which includes 482 genes known to cause a broad range of different types of immunodeficiencies, including increased susceptibility to viral infections and viral encephalitis (S2). The filtration was focused on rare (Variant Allel Frequency < 0.01) variants in coding sequence or splice regions, both single-nucleotide variants (SNV) and indels (insertions/deletions), as well as structural variants (including copy number variation). In the list of variants detected after filtration, none were considered able to explain the phenotype of the patient. The genetic investigation was further broadened to include previously polymorphisms linked to increased susceptibility to viral infection, particularly TBE encephalitis [19] (supp table S12). All detected variants are common in healthy populations (10–60%), and their contribution to disease in the patient is thereby difficult to assess. General statistics from the sequencing and all variants detected after filtration are summarised in supplementary tables (S1, S4–S7).

Furthermore, genes connected to increased intracranial pressure, using HPO (Human Phenotype Ontology) term HP:0002516, were investigated, focusing on rare variants in exonic/splice regions (S3, S8–S11).

In summary, no genetic variant could be identified in the patient that could explain the severe clinical course and fatal outcome.

### Viral genome sequencing and bioinformatics

A tiled PCR system was developed based on existing sequences to cover the whole TBE genome, utilising the primer scheme design principles described by Quick et al. [20]. Following initial amplification, sequencing libraries were prepared using the Native Barcoding Kit 24 V14 (Oxford Nanopore Technologies). Sequencing was performed on the GridION platform using an R10.4.1 flow cell.

Raw data processing was conducted as follows: Initial Quality Control was performed using nanoq [21], filtering reads shorter than 100 bp and longer than 5000 bp. Post-filtering, reads were mapped to the reference genome (GCF_000863125.1) using minimap2 [22]. Primer sequences were masked using samtools ampliconclip, and consensus sequences were generated using samtools [23]. 

#### Sequence selection and alignment

All available TBEV sequences (*n* = 161) were retrieved from the National Centre for Biotechnology Information (NCBI) Viruses database. Metadata, including accession numbers, organism specifics, sequence length, and submission dates, were extracted using Biopython [24] and are listed in Supplementary Table S13. Sequences were selected to represent the known global diversity of TBEV, including the European, Siberian, and Far-Eastern subtypes. Initial multiple sequence alignment was performed using MAFFT (Multiple Alignment using Fast Fourier Transform) [25]. To determine the evolutionary position of the newly sequenced strain, the prototype sequence was aligned towards the existing 161-sequence alignment, facilitating robust phylogenetic placement.

#### Phylogenetic inference

Phylogenetic reconstruction was performed using the Maximum Likelihood (ML) method implemented in IQ-TREE (version 3.0.1) [26]. Model selection was conducted automatically using ModelFinder [27], which identified the General Time Reversible model with empirical base frequencies, a proportion of invariant sites, and a FreeRate model with three categories (GTR+F+I+R3) as the best-fit substitution model based on the Bayesian Information Criterion (BIC).

The analysis utilised 11,685 nucleotide sites, of which 4,054 were parsimony-informative. Tree topology was inferred under the selected model, with branch support assessed using standard likelihood metrics. The final tree log-likelihood was −116727.61

#### Phylogenetic visualisation and analysis

Post-hoc phylogenetic analysis and visualisation were performed in the R statistical computing environment [28]. Tree data was processed using the ape and phytools packages [29]. The global phylogeny was midpoint rooted to optimise the resolution of major clades using phytools:midpoint.root.

The visualisation was generated using ggtree and cowplot for composite figure creation [30, 31]. Clades were manually annotated based on reference strains corresponding to major TBEV subtypes, including the European (e.g., Neudoerfl, Hypr), Siberian (e.g., Vasilchenko, Zausaev), and Far-Eastern (e.g., Sofjin, Oshima) variants. Additionally, Obsky, Himalayan, and Baikalian lineages were identified using key search terms mapped to tip labels. To investigate the local evolutionary history of the target strain TBEV_Swe_2023, a focused subtree analysis was performed. The ancestral node five levels distinct from the target tip was identified, and the corresponding clade was extracted using ape:extract.clade. The resulting local phylogeny was visualised to highlight the target strain’s relationship with its immediate neighbours.

**Fig. 3:**
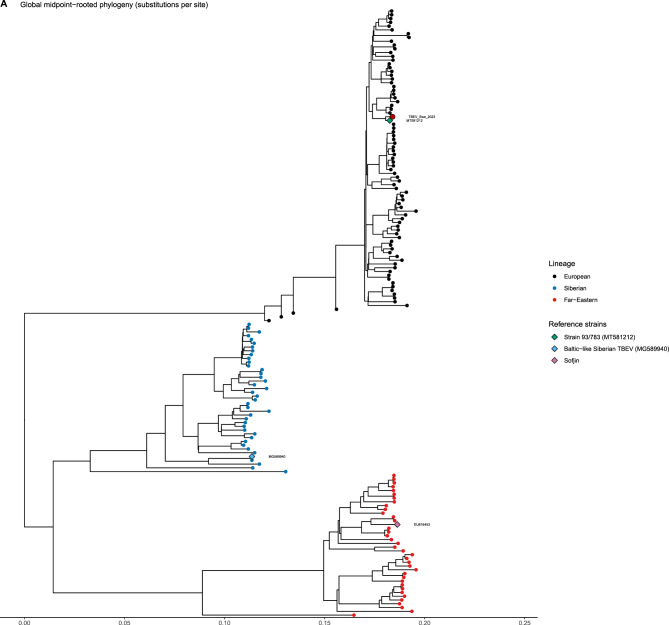

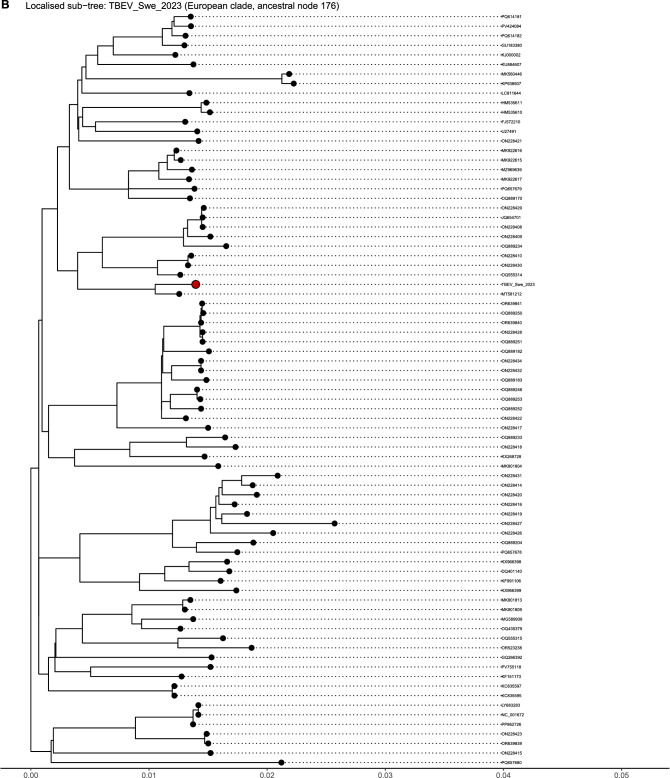
TBEV phylogeny and placement of TBEV_Swe_2023. (**A**) Global midpoint-rooted phylogeny from whole-genome alignment. The tree resolves three classical TBEV subtypes by tip colours: European (black), Siberian (blue), and Far Eastern (red). Subtype assignment follows the branching pattern. Diamonds mark reference strains: 93/783 (MT581212) for European, MG589940 for Baltic-like Siberian TBEV, and sofjin for Far Eastern. The outgroup sequence (PV568692) was dropped from the displayed ingroup. Branch lengths show nucleotide substitutions per site. A filled red point emphasises TBEV_Swe_2023. (**B**) Localised sub-tree showing TBEV_Swe_2023 and its phylogenetic neighbours. The tree shows the most recent common ancestor of TBEV_Swe_2023 and NC_001672 (the European reference, ancestral node 176). Tips: use the European colour scale. A filled red point emphasises TBEV_Swe_2023. Tip names align right, with dotted lines connecting each tip to its label. Branch lengths as in (A)

The phylogenetic analysis resulted in a tree (Fig. 3A). After performing a focused subtree analysis (Fig. 3B), the new strain was shown to be closely related to MT581212.1, a sequence previously recovered from Sweden. The two sequences share 99.46% nucleotide identity, with a difference of 0.54% across 10,425 comparable sites.

## Discussion

We report a rare case of fatal fulminant encephalitis caused by TBEV in a previously healthy child. The case is unusual in several aspects: the atypical clinical presentation, the aggressive clinical course, and the fatal outcome despite complete vaccination and no detectable immunodeficiency. The child did not present with fever. In addition, it is noteworthy that the child lacked the typical biphasic illness that is characteristic of the European subtype of TBE and reported in the majority (40–90%) of paediatric cases [1]. An association between monophasic course and severe forms of TBE has been reported as well as with vaccine failures [32, 33]. This atypical manifestation surely delayed the diagnosis and appropriate medical care at the ED. It is unfortunate that lumbar puncture was not performed at the ED on day 4 and that aggressive intravenous fluid resuscitation was administered on day 5. The limited ibuprofen exposure and its timing make a causal role in the acute deterioration unlikely. Although certain non-steroidal anti-inflammatory drugs (NSAIDs) have been associated with encephalopathy in elderly with influenza and herpes virus infections, there is no specific evidence supporting such an effect with ibuprofen in TBE [34]. There is no approved specific antiviral treatment for TBE, but therapies for cerebral oedema, such as mannitol and corticosteroids, have been used in severe cases of TBE, although no controlled studies exist [9, 14]. The use of corticosteroids is likely influenced by selection bias, as they are typically reserved for the most severe cases, complicating the assessment of their actual efficacy.

The encephalitis progressed extremely rapidly, with signs of herniation evident before external ventricular drains were inserted on the sixth day of symptoms. This aggressive clinical course is uncommon in the European subtype of TBEV, especially in children and immunocompetent individuals [8, 14]. In contrast, TBE in immunocompromised adults is associated with a relatively poor prognosis. This child had no history of recurrent or severe infections, no signs of immunodeficiency detected on extensive genetic testing using whole genome sequencing, and was seronegative for HIV.

There is conflicting data on the significance of certain polymorphisms in human genes involved in the inflammatory and antiviral response for the risk of contracting TBE or for the severity of the disease [35–38]. A non-functional chemokine receptor 5 (CCR5) has been suggested in some studies to predispose adults to develop clinical TBE [35, 36], although this finding has not been confirmed by others [37, 38]. Nonetheless, our patient did not carry the CCR5 Δ32 mutation.

In addition, the child had completed a full primary immunization against TBE. However, we cannot determine whether this child had protective antibody levels, as no stored pre-illness blood was available for analysis of neutralizing antibodies. The measured IgG levels during the CNS phase of the disease likely represent a combination of vaccine- and infection-induced antibodies and, although the assay was qualitative, there was no indication of low antibody levels.

A booster vaccination is recommended three years after the primary immunization and thereafter every 5–10 years for individuals under 50 years of age. In this case 3.5 years had passed without a booster dose. Breakthrough infections after TBE vaccination are mostly reported in elderly who started vaccination after the age of 50 years and in immunocompromised individuals, but may occur also in children [33, 39, 40]. In a study of patients hospitalized with TBE in Austria, a higher proportion of severe TBE disease was observed in vaccinated individuals, especially in children, compared to unvaccinated individuals [40]. This does not reflect a higher risk associated with vaccination but a lower field effectiveness against severe than mild disease, 82.7% versus 95.6% for children aged 1–16 years [40]. Based on these data, the authors recommended a change in the TBE vaccination schedule in Austria by implementing an extra priming dose for those given the paediatric vaccine dose or by reintroducing the adult formulation for children.

The absence of predisposing risk factors for severe disease raised suspicion that the encephalitis could have been caused by a novel TBEV strain in this area that may have a more aggressive clinical course. In order to investigate this and with the aim to recover the complete genome of the TBEV causing the infection, we used a previously unpublished tiled amplicon methodology, which, with similar protocols, has been proven effective for recovering near-complete genomes for molecular epidemiology and evolutionary studies of a range of viruses [41–45]. The TBEV genome was recovered from brain tissue obtained at autopsy and found to be a previously known European subtype. This subtype should be covered by the vaccines the child had received. The sequencing and subsequent analyses indicate that the TBEV from the child was closely related to previously recovered sequences from Sweden.

The European subtype of TBE is endemic in Sweden and is spreading across an increasingly large area that covers almost half of the country. There is a seasonal variation, with most cases reported in August and September due to varying tick activity depending on the temperature. The incidence of TBE has increased significantly by an average of ten percent per year since 2014 and reached 5.6 cases per 100,000 inhabitants in Sweden in 2023, which was the highest annual number since the disease became notifiable [2]. The annual TBE infection incidence in the area where the patient is reported to have been infected is even higher, 9.1 cases per 100,000 inhabitants [3]. As TBE incidence increases, a corresponding rise in severe cases is expected.

The diagnosis of TBE in immunocompetent individuals is dependent on serology since TBEV can typically no longer be detected by PCR in blood, cerebrospinal fluid, or urine at the onset of CNS symptoms [14]. Since vaccinated individuals are expected to have anti-TBE IgG in their serum, intrathecal antibody detection has been required for diagnosis. Here we show the utility of the detection of antibodies in serum towards TBE antigens not found in current vaccines, that is, the NS1 antigen [17].

In conclusion, this case shows that immunocompetent and vaccinated children may also develop severe and fatal encephalitis caused by the European subtype of TBEV. Atypical presentations can delay diagnosis and treatment, underscoring the need to consider TBE in the differential diagnosis of headaches and neurological symptoms in highly endemic areas, regardless of vaccination status or the presence of fever. Vaccination against TBE remains safe and effective in reducing the risk of severe disease and is vital for children in high-risk regions. Finally, antibody testing for the TBE NS1 antigen is a valuable diagnostic tool in vaccinated individuals.

## Electronic supplementary material

Below is the link to the electronic supplementary material.

Supplementary material 1

Supplementary material 2

## Data Availability

Sequencing data and assembly are available at the European Nucleotide Archive, under the study accession PRJEB106150, Biosample accession ERS30341827. Primer sequences are available upon request.

## Abbreviations

CNS: Central nervous system
CRP: C-reactive protein
CSF: Cerebrospinal fluid
CT: Computed tomography
DNA: Deoxyribonucleic acid
EBV: Epstein-Barr virus
ED: Emergency Department
Hb: Haemoglobin
HIV: Human immunodeficiency virus
HHV-6: Human herpes virus 6
HPO: Human Phenotype Ontology
ICP: Intracranial pressure
Iv: Intravenous
IVIG: Intravenous immunoglobulins
MAFFT: Multiple Alignment using Fast Fourier Transform
ML: Maximum Likelihood
NCBI: National Center for Biotechnology Information
NS1: Non-structural protein 1
NSAID: Non-steroidal anti-inflammatory drug
PCR: Polymerase Chain Reaction
SMIA: Suspension Multiplex Immunoassay
SNV: Single-nucleotide variants
TBE: Tick-borne encephalitis
TBEV: Tick-borne encephalitis virus
WBC: White blood cell
WGS: Whole-genome sequencing
