# Supplementary material for: Fatal European subtype tick-borne encephalitis in a fully vaccinated immunocompetent child: a case report with viral sequencing
Source: BMC Infect Dis. 2026 May 29;26:1048. doi: 10.1186/s12879-026-13657-0 (PMC13221759; doi:10.1186/s12879-026-13657-0)
Supplement: Supplementary file 2 — Supplementary material 2 [file 12879_2026_13657_MOESM2_ESM.docx]

| Accession | Description | Create Date | Update Date |
| --- | --- | --- | --- |
| PV568692 | Tick-borne encephalitis virus isolate selenge polyprotein gene, complete cds | 2025-05-27 | 2025-05-27 |
| ON228408 | Tick-borne encephalitis virus isolate R1672 polyprotein gene, complete cds | 2022-07-11 | 2022-07-11 |
| NC_001672 | Tick-borne encephalitis virus, complete genome | 1995-09-05 | 2019-05-23 |
| LY683283 | KR 1020190039140-A/5: COMPOSITIONS AND METHODS FOR FLAVIVIRUS VACCINATION | 2019-12-04 | 2019-12-04 |
| U27491 | Tick-borne encephalitis virus 263 polyprotein, complete cds | 1995-09-02 | 1996-02-03 |
| OR523238 | Tick-borne encephalitis virus isolate EU-Switzerland-2019-Thurgau, complete genome | 2023-10-16 | 2023-10-16 |
| KC835595 | Tick-borne encephalitis virus strain 114, complete genome | 2014-01-07 | 2014-01-07 |
| MN542364 | Tick-borne encephalitis virus strain Rus/Ix_persulcatus/Karelia/2/2018 polyprotein gene, complete cds | 2020-11-22 | 2020-11-22 |
| OP902894 | Tick-borne encephalitis virus isolate C11/13(mouse), complete genome | 2023-05-27 | 2023-05-27 |
| OP902895 | Tick-borne encephalitis virus isolate C11/13-8m, complete genome | 2023-05-27 | 2023-05-27 |
| MH645618 | Tick-borne encephalitis virus strain TBEV-2836, complete genome | 2020-01-07 | 2020-01-07 |
| MH645619 | Tick-borne encephalitis virus strain TBEV-2922, complete genome | 2020-01-07 | 2020-01-07 |
| MF398818 | UNVERIFIED: Tick-borne encephalitis virus isolate JLCB11-08 genomic sequence | 2018-05-26 | 2018-05-26 |
| MF398819 | UNVERIFIED: Tick-borne encephalitis virus isolate JLCB11-35 genomic sequence | 2018-05-26 | 2018-05-26 |
| LC017692 | Tick-borne encephalitis virus RNA, complete genome, strain: MGL-Selenge-13-12 | 2015-02-04 | 2015-02-04 |
| LC017693 | Tick-borne encephalitis virus RNA, complete genome, strain: MGL-Selenge-13-14 | 2015-02-04 | 2015-02-04 |
| MN114635 | Tick-borne encephalitis virus strain TSA-18, complete genome | 2020-08-05 | 2020-08-17 |
| MN114637 | Tick-borne encephalitis virus strain 1827-18, complete genome | 2020-08-05 | 2020-08-05 |
| MN615727 | Tick-borne encephalitis virus strain HLB-T74 polyprotein gene, complete cds | 2020-07-28 | 2020-07-28 |
| MF398820 | UNVERIFIED: Tick-borne encephalitis virus isolate JLCB11-40 genomic sequence | 2018-05-26 | 2018-05-26 |
| OR792463 | Tick-borne encephalitis virus isolate ANE-FE-2021 polyprotein gene, complete cds | 2024-12-17 | 2024-12-17 |
| KP716974 | Tick-borne encephalitis virus clone Hypr_IC, complete genome | 2015-07-12 | 2015-07-12 |
| KP716976 | Tick-borne encephalitis virus clone Hypr[Vs_E], complete genome | 2015-07-12 | 2015-07-12 |
| KP716977 | Tick-borne encephalitis virus clone Hypr[Vs_prM-E], complete genome | 2015-07-12 | 2015-07-12 |
| KP716978 | Tick-borne encephalitis virus clone Hypr[Vs_str], complete genome | 2015-07-12 | 2015-07-12 |
| JX534167 | Tick-borne encephalitis virus isolate Xinjiang-01, complete genome | 2012-10-30 | 2012-10-30 |
| LC440460 | Tick-borne encephalitis virus Nanporo-18-44 RNA, complete genome | 2018-12-22 | 2018-12-22 |
| MF774565 | Tick-borne encephalitis virus isolate TBEV-2871, complete genome | 2017-11-05 | 2017-12-13 |
| GU121642 | Tick-borne encephalitis virus strain Svetlogorie, complete genome | 2010-08-09 | 2010-08-09 |
| LC440459 | Tick-borne encephalitis virus Sapporo-17-Io1 RNA, complete genome | 2018-12-22 | 2018-12-22 |
| AB753012 | Tick-borne encephalitis virus RNA, complete genome, strain: Oshima 08-As | 2012-10-04 | 2012-10-04 |
| AB062063 | Tick-borne encephalitis virus gene, complete cds, strain:Oshima 5-10 | 2001-11-10 | 2004-09-29 |
| EU816453 | Tick-borne encephalitis virus strain Primorye-69, complete genome | 2008-07-16 | 2008-11-18 |
| KX268728 | Tick-borne encephalitis virus strain MucAr-HB-171/11, complete genome | 2016-06-28 | 2016-06-28 |
| JQ825149 | Tick-borne encephalitis virus strain Primorye-87 polyprotein gene, complete cds | 2012-08-06 | 2013-07-09 |
| OR792465 | Tick-borne encephalitis virus isolate ANE-FE-2022 polyprotein gene, complete cds | 2024-12-17 | 2024-12-17 |
| MT581212 | Tick-borne encephalitis virus strain 93/783, complete genome | 2020-10-12 | 2020-10-12 |
| KJ000002 | Tick-borne encephalitis virus strain Absettarov, complete genome | 2014-03-19 | 2014-03-19 |
| KC835597 | Tick-borne encephalitis virus strain CGl223, complete genome | 2014-01-07 | 2014-01-07 |
| KF151173 | Tick-borne encephalitis virus strain A104, complete genome | 2013-07-08 | 2013-08-14 |
| GU183380 | Tick-borne encephalitis virus strain Kumlinge A52 polyprotein gene, complete cds | 2012-01-05 | 2012-03-21 |
| HM535610 | Tick-borne encephalitis virus strain KrM 213, complete genome | 2011-03-07 | 2011-06-14 |
| HM535611 | Tick-borne encephalitis virus strain KrM 93, complete genome | 2011-03-07 | 2011-06-14 |
| HM859894 | Tick-borne encephalitis virus strain Primorye-633, complete genome | 2010-12-21 | 2010-12-21 |
| HM859895 | Tick-borne encephalitis virus strain Primorye-2239, complete genome | 2010-12-21 | 2010-12-21 |
| GQ266392 | Tick-borne encephalitis virus isolate AS33, complete genome | 2010-04-15 | 2010-04-15 |
| DQ401140 | Tick-borne encephalitis virus isolate Toro-2003, complete genome | 2006-12-11 | 2013-10-23 |
| JQ825157 | Tick-borne encephalitis virus strain Primorye-202 polyprotein gene, complete cds | 2012-08-06 | 2013-07-09 |
| GQ228395 | Tick-borne encephalitis virus strain Primorye-18, complete genome | 2009-07-14 | 2009-07-14 |
| PQ657680 | Tick-borne encephalitis virus, complete genome | 2025-02-01 | 2025-02-01 |
| MN615726 | Tick-borne encephalitis virus strain JL-T75 polyprotein gene, complete cds | 2020-07-28 | 2020-07-28 |
| MN615728 | Tick-borne encephalitis virus strain DXAL-T83 polyprotein gene, complete cds | 2020-07-28 | 2020-07-28 |
| PV683036 | Tick-borne encephalitis virus isolate 24_2_K84_3 polyprotein gene, complete cds | 2025-06-02 | 2025-06-02 |
| PQ657678 | Tick-borne encephalitis virus, complete genome | 2025-02-01 | 2025-02-01 |
| OQ555314 | Tick-borne encephalitis virus isolate GR_RF/Switzerland/2021 polyprotein gene, complete cds | 2023-08-23 | 2024-02-02 |
| OQ889251 | Tick-borne encephalitis virus isolate 2008-P3-S9 polyprotein-like gene, complete sequence | 2023-08-15 | 2023-08-15 |
| MT344092 | Tick-borne encephalitis virus strain 1776, complete genome | 2023-03-30 | 2023-03-30 |
| JQ654701 | Tick-borne encephalitis virus strain Ljubljana I polyprotein gene, complete cds | 2012-12-04 | 2012-12-04 |
| ON228429 | Tick-borne encephalitis virus isolate R1672 polyprotein gene, complete cds | 2022-07-11 | 2022-07-11 |
| MG589939 | Tick-borne encephalitis virus strain Kuutsalo_2_Human_Cerebellum_Finland-2015, complete genome | 2018-04-29 | 2018-04-29 |
| MG589940 | Tick-borne encephalitis virus strain Kotka-18_Ixodes_ricinus_Finland-2011, complete genome | 2018-04-29 | 2018-04-29 |
| KP938507 | Tick-borne encephalitis virus strain Sorex 18-10, complete genome | 2015-05-04 | 2015-06-23 |
| OR792466 | Tick-borne encephalitis virus isolate CY-FE-2022 polyprotein gene, complete cds | 2024-12-17 | 2024-12-17 |
| PQ553684 | Tick-borne encephalitis virus isolate D-TBEV-XAM polymerase gene, complete cds | 2024-12-11 | 2024-12-11 |
| PQ790055 | Tick-borne encephalitis virus strain HLB-H197 polyprotein gene, complete cds | 2025-06-26 | 2025-06-26 |
| PQ790051 | Tick-borne encephalitis virus strain HLB-ZG50 polyprotein gene, complete cds | 2025-06-26 | 2025-06-26 |
| PQ790056 | Tick-borne encephalitis virus strain HLB-H372 polyprotein gene, complete cds | 2025-06-26 | 2025-06-26 |
| PQ790052 | Tick-borne encephalitis virus strain HLB-ZH141 polyprotein gene, complete cds | 2025-06-26 | 2025-06-26 |
| OR896869 | Tick-borne encephalitis virus isolate 23-Kyr-KDCA-26, complete genome | 2024-01-07 | 2024-01-07 |
| OQ889253 | Tick-borne encephalitis virus isolate 2021-P112_S3 polyprotein-like gene, complete sequence | 2023-08-15 | 2023-08-15 |
| ON675587 | Tick-borne encephalitis virus isolate Novososedovo_1, complete genome | 2023-02-22 | 2023-02-22 |
| PQ790053 | Tick-borne encephalitis virus strain HLB-ZH148 polyprotein gene, complete cds | 2025-06-26 | 2025-06-26 |
| PQ790054 | Tick-borne encephalitis virus strain HLB-H168 polyprotein gene, complete cds | 2025-06-26 | 2025-06-26 |
| KF991106 | Tick-borne encephalitis virus strain Saringe-2009 polyprotein gene, complete cds | 2014-07-28 | 2014-07-28 |
| PQ790057 | Tick-borne encephalitis virus strain ARS-H457 polyprotein gene, complete cds | 2025-06-26 | 2025-06-26 |
| OQ435379 | Tick-borne encephalitis virus strain 493, complete genome | 2023-07-04 | 2023-07-04 |
| MG589938 | Tick-borne encephalitis virus strain Kuutsalo-14_Ixodes_ricinus_Finland-2017, complete genome | 2018-04-29 | 2018-04-29 |
| KX966398 | Tick-borne encephalitis virus strain JP-296, complete genome | 2017-01-02 | 2017-01-02 |
| KX966399 | Tick-borne encephalitis virus strain JP-554, complete genome | 2017-01-02 | 2017-01-02 |
| FJ572210 | Tick-borne encephalitis virus strain Salem, complete genome | 2009-01-21 | 2009-01-21 |
| PV424084 | Tick-borne encephalitis virus isolate Larvik_2024, partial genome | 2025-07-21 | 2025-07-21 |
| PQ657679 | Tick-borne encephalitis virus, complete genome | 2025-02-01 | 2025-02-01 |
| ON228432 | Tick-borne encephalitis virus isolate 11599 polyprotein gene, complete cds | 2022-07-11 | 2022-07-11 |
| KT001073 | Tick-borne encephalitis virus strain Lazo MP36, complete genome | 2015-08-24 | 2015-08-24 |
| PQ614181 | Tick-borne encephalitis virus isolate Larvik-4_2021, partial genome | 2025-07-14 | 2025-07-14 |
| PQ614182 | Tick-borne encephalitis virus isolate Sandoya-1_2022, partial genome | 2025-07-14 | 2025-07-14 |
| ON228430 | Tick-borne encephalitis virus isolate NK6108 polyprotein gene, complete cds | 2022-07-11 | 2022-07-11 |
| KF880803 | Tick-borne encephalitis virus strain 9024 from Russia polyprotein-precursor, gene, complete cds | 2013-12-18 | 2013-12-18 |
| MK922615 | Tick-borne encephalitis virus strain HB_IF06_8033 polyprotein gene, complete cds | 2019-06-16 | 2019-06-16 |
| MK922617 | Tick-borne encephalitis virus strain Rauher_BuschP19_S40 polyprotein gene, complete cds | 2019-06-16 | 2019-06-16 |
| MK922616 | Tick-borne encephalitis virus strain HB_IF06_8040 polyprotein gene, complete cds | 2019-06-16 | 2019-06-16 |
| PV683035 | Tick-borne encephalitis virus isolate 24_2_K86_1 polyprotein gene, complete cds | 2025-06-02 | 2025-06-02 |
| PV568693 | Tick-borne encephalitis virus isolate aershan polyprotein gene, complete cds | 2025-05-27 | 2025-05-27 |
| PP862726 | Tick-borne encephalitis virus isolate MBC045, partial genome | 2024-07-08 | 2024-07-08 |
| KP844727 | Tick-borne encephalitis virus strain Birobidzhan 1357, complete genome | 2015-04-21 | 2015-04-21 |
| KF880805 | Tick-borne encephalitis virus strain 1230 from Russia polyprotein-precursor, gene, complete cds | 2013-12-18 | 2013-12-18 |
| ON228428 | Tick-borne encephalitis virus isolate R932 polyprotein gene, complete cds | 2022-07-11 | 2022-07-11 |
| ON228434 | Tick-borne encephalitis virus isolate 11599 polyprotein gene, complete cds | 2022-07-11 | 2022-07-11 |
| KP844724 | Tick-borne encephalitis virus strain Chichagovka 1222, complete genome | 2015-04-21 | 2015-04-21 |
| KP844725 | Tick-borne encephalitis virus strain Chichagovka 1223, complete genome | 2015-04-21 | 2015-04-21 |
| MK560446 | Tick-borne encephalitis virus strain 172-68, complete genome | 2021-03-04 | 2021-03-04 |
| MT246197 | Tick-borne encephalitis virus isolate JL_Jiaohe polyprotein gene, complete cds | 2020-07-18 | 2020-07-18 |
| MF374487 | Tick-borne encephalitis virus strain Oshima 5.10 polyprotein gene, complete cds | 2018-05-14 | 2018-05-14 |
| KT001070 | Tick-borne encephalitis virus strain Khekhtzir 9-13, complete genome | 2015-08-24 | 2015-08-24 |
| KT001072 | Tick-borne encephalitis virus strain Khekhtzir 17-13, complete genome | 2015-08-24 | 2015-08-24 |
| KP844726 | Tick-borne encephalitis virus strain Birobidzhan 1354, complete genome | 2015-04-21 | 2015-04-21 |
| PV755118 | Tick-borne encephalitis virus isolate TBEV/168/2025, complete genome | 2025-06-11 | 2025-06-11 |
| PV683043 | Tick-borne encephalitis virus isolate 24_2_K16_6 polyprotein gene, complete cds | 2025-06-02 | 2025-06-02 |
| ON408071 | Tick-borne encephalitis virus strain NE-TH3, complete genome | 2022-10-11 | 2022-10-11 |
| ON408072 | Tick-borne encephalitis virus strain NE-TH4, complete genome | 2022-10-11 | 2022-10-11 |
| ON408073 | Tick-borne encephalitis virus strain NE-SL4, complete genome | 2022-10-11 | 2022-10-11 |
| KU884607 | Tick-borne encephalitis virus isolate Leila-BH95/15, partial genome | 2016-03-21 | 2016-03-21 |
| PV683042 | Tick-borne encephalitis virus isolate 24_2_K19_4 polyprotein gene, complete cds | 2025-06-02 | 2025-06-02 |
| PV626568 | MAG: Tick-borne encephalitis virus isolate TBEV-IP-1, complete genome | 2025-05-20 | 2025-05-20 |
| OR827302 | Tick-borne encephalitis virus isolate IM-Sib-2020 polyprotein gene, complete cds | 2024-12-17 | 2024-12-17 |
| JN003209 | Tick-borne encephalitis virus strain Irkutsk-12 polyprotein gene, complete cds | 2011-10-24 | 2012-11-05 |
| OQ889246 | Tick-borne encephalitis virus isolate 2021-P112-S1 polyprotein-like gene, complete sequence | 2023-08-15 | 2023-08-15 |
| PQ553685 | Tick-borne encephalitis virus isolate K-TBEV-HL polymerase gene, complete cds | 2024-12-11 | 2024-12-11 |
| OQ555315 | Tick-borne encephalitis virus isolate ZHw_UM/Switzerland/2022 polyprotein gene, complete cds | 2023-08-23 | 2024-02-02 |
| OQ889182 | Tick-borne encephalitis virus isolate 2009-P75-S1 polyprotein-like gene, complete sequence | 2023-08-15 | 2023-08-15 |
| PV683041 | Tick-borne encephalitis virus isolate 24_2_K28_3 polyprotein gene, complete cds | 2025-06-02 | 2025-06-02 |
| PV788233 | Tick-borne encephalitis virus isolate MN-TBEV-24-013, complete genome | 2025-06-29 | 2025-11-03 |
| PV788234 | Tick-borne encephalitis virus isolate MN-TBEV-24-014, complete genome | 2025-06-29 | 2025-11-03 |
| PV683025 | Tick-borne encephalitis virus isolate 24_2_K44_3 polyprotein gene, complete cds | 2025-06-02 | 2025-06-02 |
| JN003208 | Tick-borne encephalitis virus strain Cht-22 polyprotein gene, complete cds | 2011-10-24 | 2012-11-05 |
| PV173737 | Tick-borne encephalitis virus isolate Likeysa 9-24, complete genome | 2025-03-16 | 2025-03-16 |
| MK801809 | Tick-borne encephalitis virus isolate E266-Espoo-Finland-2017, complete genome | 2019-06-15 | 2019-06-15 |
| OQ889252 | Tick-borne encephalitis virus isolate 2008-P54-S4 polyprotein gene, partial cds | 2023-08-15 | 2023-08-15 |
| MN114636 | Tick-borne encephalitis virus strain 1512-18, complete genome | 2020-08-05 | 2020-08-05 |
| OQ889183 | Tick-borne encephalitis virus isolate 2009-P87-S1 polyprotein-like gene, partial sequence | 2023-08-15 | 2023-08-15 |
| PV626569 | MAG: Tick-borne encephalitis virus isolate TBEV-IP-2, complete genome | 2025-05-20 | 2025-05-20 |
| PV683030 | Tick-borne encephalitis virus isolate 24_2_K23_2 polyprotein gene, complete cds | 2025-06-02 | 2025-06-02 |
| LC811644 | Tick-borne encephalitis virus NLEar_322 RNA, nearly complete genome | 2025-05-02 | 2025-05-02 |
| PV683024 | Tick-borne encephalitis virus isolate 24_2_K54_1 polyprotein gene, complete cds | 2025-06-02 | 2025-06-02 |
| PV683031 | Tick-borne encephalitis virus isolate 24_2_K25_2 polyprotein gene, complete cds | 2025-06-02 | 2025-06-02 |
| OR639839 | Tick-borne encephalitis virus isolate 2018-P29-S2 polyprotein gene, complete cds | 2024-12-09 | 2024-12-09 |
| OQ889250 | Tick-borne encephalitis virus isolate 2008-P3-S8 polyprotein-like gene, complete sequence | 2023-08-15 | 2023-08-15 |
| ON228409 | Tick-borne encephalitis virus isolate 5171 polyprotein gene, complete cds | 2022-07-11 | 2022-07-11 |
| MK801813 | Tick-borne encephalitis virus isolate K15-Espoo-Finland-2018, complete genome | 2019-06-15 | 2019-06-15 |
| OQ889204 | Tick-borne encephalitis virus isolate 2014-P101-S1 polyprotein-like gene, complete sequence | 2023-08-15 | 2023-08-15 |
| ON228418 | Tick-borne encephalitis virus isolate R5685 polyprotein gene, complete cds | 2022-07-11 | 2022-07-11 |
| ON228427 | UNVERIFIED: Tick-borne encephalitis virus isolate NK2696 polyprotein-like gene, complete sequence | 2022-07-11 | 2022-07-11 |
| OR639840 | Tick-borne encephalitis virus isolate 2018-P29-S3 polyprotein gene, complete cds | 2024-12-09 | 2024-12-09 |
| OR639841 | Tick-borne encephalitis virus isolate 2018-P45-S3 polyprotein gene, complete cds | 2024-12-09 | 2024-12-09 |
| ON228417 | UNVERIFIED: Tick-borne encephalitis virus isolate R5837 polyprotein-like gene, complete sequence | 2022-07-11 | 2022-07-11 |
| ON228426 | UNVERIFIED: Tick-borne encephalitis virus isolate NK8558 polyprotein-like gene, partial sequence | 2022-07-11 | 2022-07-11 |
| ON228410 | Tick-borne encephalitis virus isolate NK6108 polyprotein gene, complete cds | 2022-07-11 | 2022-07-11 |
| ON228414 | Tick-borne encephalitis virus isolate NK4357 polyprotein gene, complete cds | 2022-07-11 | 2022-07-11 |
| ON228415 | Tick-borne encephalitis virus isolate R5799 polyprotein gene, complete cds | 2022-07-11 | 2022-07-11 |
| ON228419 | Tick-borne encephalitis virus isolate R5843 polyprotein gene, complete cds | 2022-07-11 | 2022-07-11 |
| ON228420 | Tick-borne encephalitis virus isolate 12340 polyprotein gene, complete cds | 2022-07-11 | 2022-07-11 |
| ON228421 | Tick-borne encephalitis virus isolate NK8568 polyprotein gene, complete cds | 2022-07-11 | 2022-07-11 |
| ON228423 | UNVERIFIED: Tick-borne encephalitis virus isolate NK8556 polyprotein-like gene, complete sequence | 2022-07-11 | 2022-07-11 |
| ON228416 | Tick-borne encephalitis virus isolate R2758 polyprotein gene, complete cds | 2022-07-11 | 2022-07-11 |
| ON228422 | UNVERIFIED: Tick-borne encephalitis virus isolate R2582 polyprotein-like gene, partial sequence | 2022-07-11 | 2022-07-11 |
| ON228431 | UNVERIFIED: Tick-borne encephalitis virus isolate NK4357 polyprotein-like gene, complete sequence | 2022-07-11 | 2022-07-11 |
| OQ889170 | Tick-borne encephalitis virus isolate 2008-P110-S4 polyprotein-like gene, complete sequence | 2023-08-15 | 2023-08-15 |
| MZ969639 | Tick-borne encephalitis virus isolate NL-RMB2 polyprotein gene, complete cds | 2022-12-01 | 2022-12-01 |
| OQ889233 | Tick-borne encephalitis virus isolate 2018-P106-S1 polyprotein-like gene, complete sequence | 2023-08-15 | 2023-08-15 |
| OQ889234 | Tick-borne encephalitis virus isolate 2019-P107-S3 polyprotein-like gene, complete sequence | 2023-08-15 | 2023-08-15 |
| MK801804 | Tick-borne encephalitis virus isolate Sipoo-4-Finland-2013, complete genome | 2019-06-15 | 2019-06-15 |

Supplementary Table S13. GenBank accession numbers and associated metadata for Tick-borne encephalitis virus (TBEV) sequences. This dataset comprises 161 sequences used in this study. Data was retrieved from the NCBI Nucleotide database. Columns include the Accession ID, sequence definition (Description), organism, sequence length (bp), taxonomic ID, and submission dates.
